# The first report on CFTR mutations of meconium ileus in cystic fibrosis population in Saudi Arabia: A single center review

**DOI:** 10.1016/j.ijpam.2021.03.008

**Published:** 2021-03-22

**Authors:** Hanaa Banjar, Raef Qeretli, Ali Ramadan, Abdullah Al-Ibraheem, Fahad Bnatig

**Affiliations:** a Department of Pediatrics, King Specialist Hospital and Research Center (KFSHRC), Riyadh, Saudi Arabia; b Department of Pediatrics, Qatif Central Hospital, Qatif, Saudi Arabia; c College of Medicine, AlFaisal University, Riyadh, Saudi Arabia

**Keywords:** Meconium ileus, Arabs, CFTR, Cystic fibrosis

## Abstract

**Introduction:**

Meconium ileus (MI) is one of the most common causes of intestinal obstruction in newborns. It is the earliest clinical manifestation of cystic fibrosis (CF). MI is suspected if a baby fails to pass meconium shortly after birth and develops symptoms of bowel obstruction, such as distention of the abdomen or vomiting. MI can lead to bowel perforation, a twisting of the bowel, or inflammation and infection of the abdominal cavity.

**Objectives:**

To find the incidence and prevalence of meconium ileus in cystic fibrosis patients and to report on the most common gene mutation of MI in CF patients.

**Methodology:**

Retrospective review of the medical documentations of all MI patients during the period of 1989–2018.

**Results:**

A total of 40 CF confirmed patients were presented with MI. Twenty-nine patients (71%) are alive and 11 patients (29%) died or lost to follow-up. The following CFTR mutations were found: Eight patients (20%) with c.2988+1G>A; Intron 18. Seven patients (17.5%) with c.1418delG; Exon 11. Five patients (12.5%) with c.579+1G>T; Intron 5. Four patients (10%) with c.1911delG; Exon 14. Four patients (10%) with c.1521_1523delCTT; Exon 11. Four patients (10%) with c.416A>T; Exon 13. Three patients (7.5%) with c.2421A>G; Exon 14. Two patients (5%) with c.3908A>C; Exon 21. One patient (2.5%) with c.3889dupT; Exon 24. One patient (2.5%) with c.1657C>T; Exon 12. One patient (2.5%) with c.2547C>A; Exon 14a. Eighteen patients (45%) were presented with vomiting, 38 patients (95%) had postnatal radiological findings, 7 patients (17.5%) had electrolytes imbalance. Five patients (12.5%) had cholestasis and 4 patients (10%) developed chronic liver disease. Thirty-five patients (79.5%) underwent surgical repair and 9 patients (20.5%) were treated medically. Mean age of operation was 2.25 (2) days. Of 9 patients, 6 (66.6%) were treated with gastrograffin enema, 2 patients (22.2%) with oral N-acetylcysteine and 1 patient (11.1%) with saline rectal wash. Thirteen patients (31.5%) required TPN. Five patients had recurrent operation.

**Conclusion:**

CF and meconium ileus are commonly present in CF patients in Saudi Arabia. Prognosis is similar to other CFs without MI, if treated early. Thirty percent of our CF/MI patients have intronic mutations.

## 1 Introduction

Cystic fibrosis (CF) is an autosomal recessive disorder caused by the alteration of a gene located on the long arm of chromosome 7 that encodes for a protein of 1480 amino acids, the cystic fibrosis transmembrane conductance regulator (CFTR), which functions as a chloride channel on the apical membrane of epithelial cells [1]. This alteration results in a change of the viscosity of secretions, and the production of thick mucus that leads to malabsorption, loss of electrolytes in sweat, and alteration of pulmonary secretions. There are more than 2000 known genetic mutations, and disease modifying genes [2]. The classic presentation of CF is chronic lung disease (recurrent pulmonary infections), exocrine pancreatic insufficiency (diarrhea and malnutrition), loss of salt, and obstructive azoospermia syndrome [3].

Meconium ileus (MI) is defined as an intestinal obstruction with thick meconium in the newborn period that occurs in 15–20% of CF patients [4,5]. Affected newborns often exhibit severe bowel obstruction caused by thickened mucous and meconium occluding the mid- or distal-part of the small bowel [6,7]. Resolution of the ileal obstruction requires extensive treatment, including rectal infusion of gastrografin and/or enema under fluoroscopy, and additional saline or acetylcysteine enemas. In many of the newborns diagnosed with CF presenting with MI, surgical intervention is necessary to alleviate the blockage, or they develop secondary complications such as intestinal atresia or intestinal perforation. The outcome of infants with CF and MI has greatly improved, and their survival is now similar to children with CF without MI [6,7]. Previous studies reported the prevalence of MI of 14% in Canada [4,5] and Italy [5], and 20% in the US [8]. The prevalence of MI was counted in relation to the total CF patients in our institution which represented around 80% of the total CF population in Saudi Arabia.

**1.1 Objectives:** To measure the prevalence of MI in CF patients in Saudi Arabia and to identify the most common CFTR gene mutations in those patients.

## 2 Methodology

A retrospective data collection of all patients with CF, who were referred to a CF clinic from 1992 to 2018 was carried out. CF was diagnosed with a typical clinical picture of cough and sputum production. In addition, a history of CF in the immediate family, high sweat chloride test result >60 mmol/L in two subsequent samples by the Wescor quantitative method, USA, or pathologic CFTR mutations on both chromosomes were observed.

**2.1 Inclusion Criteria:** Patients with CF between the period 1989 and 2018 were reviewed. Cystic fibrosis was confirmed by gene study of 2 pathogenic CFTR mutations in both alleles and Sweat chloride test results >60 mmol/L. CF Patients with meconium ileus symptoms in the first few days of life were included.

**2.2 CFTR identification:** CFTR Gene Screen Methodology: DNA Isolation, PCR amplification of genomic DNA, mutational analysis, and sequencing methods have been described in a previous study from the same center [9]. Variant detection was performed by scoring that used a publicly available variant database for CF such as “CF Mutation Database” (http://www.genet.sickkids.on.ca/CFTR/Home.html) or (http://www.hgmd.cf.ac.uk/ac/index.php). Both variant databases provided extensive report of up-to-date sequence variants, deletions, and insertions for the CFTR gene.

### 2.1 Ethical considerations and statistical methods

Ethical approval was obtained from the research advisory committee. The Declaration of Helsinki and good clinical practice guidelines were followed. Data collection and data entry were supervised by the principal investigator. All data needed were obtained using a retrospective chart review and stored in the pediatrics research unit, which could only be accessed by the principal investigator and the assigned clinical research coordinator. The entire information of the patient was kept strictly confidential. Each patient was given a study number, and all patients’ data were entered into the designated data sheet (Excel) without any means for patient identification. The Department of Biostatistics Epidemiology and Scientific Computing (BESC) carried out the statistical analysis of the data. The frequency of events was obtained from mean (SD), with simple descriptive analysis.

## 3 Results

Of the total 354 confirmed CF patients, 40 (11%) patients were confirmed to have MI. Eighteen patients (45%) were males and 22 (55%) were females. Age at diagnosis of the CF was 10 years (S.D 8–12 years and age at follow-up was 7.5 years (S.D 2–13 years). Twenty-nine patients (71%) are alive now and 11 patients (29%) had died or lost follow-up. Twenty-three patients (57.5%) were from the Eastern Province of Saudi Arabia, 7 patients (17.5%) from the South, 5 patients (12.5%) from the North, 4 patients (10%) from the Central Province and 1 patient (2.5%) from the West. The following CFTR mutations were found (Table 1) (17–27): 8 patients (20%) with c.2988+1G>A; Intron 18.7 patients (17.5%) with c.1418delG; Exon 11.5 patients (12.5%) with c.579+1G>T; Intron 5.4 patients (10%) with c.1911delG; Exon 14.4 patients (10%) with c.1521_1523delCTT; Exon 11.4 patients (10%) with c.416A>T; Exon 13.3 patients (7.5%) with c.2421A>G; Exon 14.2 patients (5%) with c.3908A>C; Exon 21.1 patient (2.5%) with c.3889dupT; Exon 24.1 patient (2.5%) with c.1657C>T; Exon 12.1 patient (2.5%) with c.2547C>A; Exon 15 (Table 1). Thirty-two patients (80%) were delivered by normal spontaneous vaginal delivery (NSVD) and 8 patients (20%) by caesarian section. Mean sweat chloride test was recorded as 92 mmol/L (S.D 80–104 mmol/L) (Clinical presentation in Table 2). Nine patients (20.5%) had medical interventions (six patients were (66.6%) treated with gastrograffin enema, two patients (22%) with oral N-acetylcysteine and 1 patient (11%) with saline rectal wash). Thirty-five patients (79.5%) underwent surgical repair. Eight out of 35 patients (23%) had colostomy and 27 patients (77%) had ileostomy. Mean age of operation was 2.25 days. Thirteen patients (31.5%) required TPN and 5 patients required repeated operation.

**Table 1:** The most common *CFTR* mutations in CF with MI patients.

Ref	refSNP	location	Nucleotide Change	Protein Change	Count	%
10	**rs75096551**	Intron 18	c.2988+1G>A	3120+1G>A	8	20
**11**	**rs77188391**	Intron 5	c.579+1G>T	711+1G>T	5	12.5
12	**rs397508205**	Exon 11	c.1418delG	p.Gly473Glufs	7	17.5
13	**rs1554389296**	Exon 14	c.1911delG	p.Gln637Hisfs	4	10
14	**rs113993960**	Exon 11	c.1521_1523delCTT	p.Phe508delPhe	4	10
15	**rs76371115**	Exon 4	c.416A>T	p.His139Leu	4	10
16	**rs1800103**	Exon 14	c.2421A>G	p.Ile807Met	3	7.5
17	**rs397508636**	Exon 21	c.3908A>C	p.Asn1303Thr	2	5
18	**rs121908808**	Exon 24	c.3889dupT	p.Ser1297Phefs	1	2.5
19	**rs74597325**	Exon 12	c.1657C>T	p.Arg553Ter	1	2.5
20	**rs397508394**	Exon 15	c.2547C>A	p.Tyr849Ter	1	2.5

Ref = the reference number that described the mutation in the literature.

refSNP = Reference Single Nucleotide Polymorphism Database, https://www.ncbi.nlm.nih.gov/snp/.

(%) = percentages.

Count = number of patients with the mutation.

**Table 2:** Clinical Presentation of all Meconium ileus patients (Total 40 patients).

S/S	Patients (N)	%
Vomiting	18	45
Electrolytes imbalance	7	17.5
Cholestasis	5	12.5
Liver disease	4	10
Postnatal radiological findings of MI	38	95

S/S = signs and symptoms.

N= Number.

(%) = percentages.

Thirty percent of our (13 patients) CF/MI patients had intronic mutations (Introns 5 and 18) (Table 1) [[10], [11], [12], [13], [14], [15], [16], [17], [18], [19], [20]], and the other 70% of mutations were found in exon locations [[10], [11], [12], [13], [14], [15], [16], [17], [18], [19], [20]].

## 4 Discussion

Meconium ileus (MI), often the earliest clinical presentation of CF, generally occurs in about 13–27% of patients diagnosed with CF [[21], [22], [23]]. [[21], [22], [23]] In our CF population, the prevalence of MI is 11% which is almost similar to the prevalence in the reported literature.

MI is most commonly reported in CF patients with Class I–III mutations, such as F508del, G542X, W1282X, R553X, and G551D [5]. However, it is interesting to note that in our study it was found that CF gene mutation c.2988+1G>A; Intron 18 and c.1418delG; Exon 11 are the most common mutations associated with MI in Saudi Arabia, which is different from the rest of the world (Table 1) [[10], [11], [12], [13], [14], [15], [16], [17], [18], [19], [20]].

The common presentations of MI reported in the literature include: abdominal distention and vomiting in 49–91%, delayed passage of meconium for more than 24 h or lack of meconium in 36–83% and visible or palpable loops of the bowel in 15–44% [24,25] of newborns which are similar to our observation.

There is less literature information about the onset of symptoms and the moment when neonates are referred to the surgeon; however, in our patients, the mean age of surgical intervention was 2.25 days and the mean age at diagnosis of CF was 10 (14 months).

Disimpaction of the meconium requires extensive treatment through rectal infusion of gastrografin under fluoroscopy, in addition to saline/acetyl cysteine enemas (9 out of 40 patients in our study or 22.5%). Many newborns with CF presenting with MI require surgical intervention to relieve the obstruction and/or manage secondary complications [6,7]. This is evident in our study in which most of the patients (79.5%) required surgery.

Infants with MI are at higher risk of cholestasis, especially if they are receiving TPN. Leeiwen et al. [26] reported an increased incidence of cholestasis in MI patients (27.1%) in comparison to those without MI (1.2%), which is likely caused by surgical intervention and TPN [26].

Thirteen (31.5%) of our patients required TPN, which is similar to other studies, and five patients developed cholestasis. Only one patient had resolved cholestasis, while the rest developed chronic liver disease. One patient later required liver transplantation. These findings support previous reports suggesting that infants with MI have a high incidence of developing liver disease [26,27].

The improvement in medical and surgical management of MI has resulted in good prognosis for CF patients with MI that is generally similar to those CF patients without MI [21].

## 5 Conclusion

Ileus is commonly present in CF patients in Saudi Arabia. Prognosis is similar to other CFs without MI if the disease is treated early. Thirty percent of our CF/MI patients have intronic mutations.

## Ethical statement

Hanaa Banjar MD, Raef Qeretli MD, Ali Ramadan MD, Abdullah Al-Ibrahim MD, Fahad Bnatig MD.

1) This material has not been published in whole or in part elsewhere;
2) The manuscript is not currently being considered for publication in another journal;
3) All authors have been personally and actively involved in substantive work leading to the manuscript, and will hold themselves jointly and individually responsible for its content.

## Declaration of competing interest

No conflict of interest between authors.
